# Effects and possible mechanism of Ruyiping formula application to breast cancer based on network prediction

**DOI:** 10.1038/s41598-019-41243-9

**Published:** 2019-03-27

**Authors:** Rui-Fang Xie, Sheng Liu, Ming Yang, Jia-Qi Xu, Zhi-Cheng Li, Xin Zhou

**Affiliations:** 1 Longhua Hospital affiliated to Shanghai University of Traditional Chinese Medicine, Shanghai, 200032 China; 2 Surgery,Shanghai Pu Dong Hospital, Shanghai, China

## Abstract

Ruyiping (RYP), a Chinese herbal formula, can remove toxin and clear nodular, showing ability of preventing postoperative recurrence of breast cancer. In this study, network was performed to predict possible targets, genes and pathways associated with RYP and breast cancer. Thin Layer Chromatography (TLC) and High Performance Liquid Chromatography (HPLC) were used to quantitatively study RYP formula and its single herbs. MTT methods, Luciferase reporter systems, zebrafish model and western blotting were respectively adopted to verify network prediction. Results showed that the quality of RYP could be controlled and icariin could be selected as mark ingredient; RYP expressed anti-breast tumor effects, which could be associated with inhibiting expression of Transforming Growth Factor β (TGFβ), promoting cells apoptosis and anti-angiogenesis. Parts of these results were consistent with network predictions in some degree, but not all. Network can help us narrow areas, focus on crucial factors, save money as well as time, but the results predicted by network should be confirmed by further experiments.

## Introduction

Breast cancer, in women worldwide a kind of malignant tumor which seriously threatens health of females, has occupied crucial position in female’s tumors for decades’ years^1,2^. Recurrence and metastasis after operation for mammary cancer, especially transferring to viscera, is the main cause of therapeutic failure^3^.

In order to treat breast cancer, Professor De-ming Lu, a famous experienced doctor in our hospital, has summarized clinical experiences of past scholars and established a formula named as RuYiPing (RYP) (its Chinese meaning is arresting transfer of breast cancer). This formula has been used in our hospital for more than 30 years and shows good therapeutic effects on breast cancer^4^. The recipe is consisted of *Epimedium brevicornu Maxim* (YYH), *Rhizoma zedoariae* (EZ), *Iphigenia* (SCG), *Nidus Vespae* (FF) and *Holboellia fargesii Reaub* (BYZ) and mainly contains chemical pure compounds such as curcumol, icariin, colchicines, curcumin, curcudione and so on. According to the theory of Traditional Chinese Medicine (TCM), its principle of treatment is to remove toxin, dissipate nodule and eliminate swell and stagnation.

Previous pharmacological results showed that RYP could inhibit growth and metastasis of breast cancer cell lines^5–7^ and mice with mammary cancer^8,9^. It could also inhibit the expression of vascular endothelial growth factor of tumor tissue in mice which were transplanted with mammary cancer^10,11^. Further clinical research also showed that RYP formula could arrest relapse and metastasis of mammary carcinoma^12,13^. However, the mechanism still need be clarified more clearly. Therefore, the objective of this article focused on investigating the primary mechanism of RYP.

Until now, it is a substantial challenge to study RYP’s mechanism. As we know, any TCM formulae including RYP are mixtures of hundreds of chemical compounds. Unlike the majority of current drugs, which are designed to selectively act on a single target, most of the active chemical ingredients in herbs may weakly or moderately act on multiple cellular targets. Herbal formulae are formulated according to special syndromes or patterns (“ZHENG” in Chinese) instead of targeting disease as modern medicine does. These facts make it difficult to systematically investigate herbal formula mechanism using routine methods^14^.

To achieve a comprehensive understanding of the mechanisms of herbal formulae, new methods and strategies such as computational methods are urgently needed. Network pharmacology is a novel research field which is based on widely existing databases allows us to form an initial understanding of the action mechanisms within the context of systems-level interactions. Since TCM herbal formula has been considered as multi-component and multi-target therapeutics which potentially meets the demands of treating a number of complex diseases in an integrated manner, the methodologies of network pharmacology are suitable for pursuing a priori knowledge about the combination rules embedded in formula^15^. The application of network pharmacology to TCM provides new possibilities for us to screen active ingredients and relevant targets which in-turn high-light the mechanism of action^16^.

**Figure 1:**
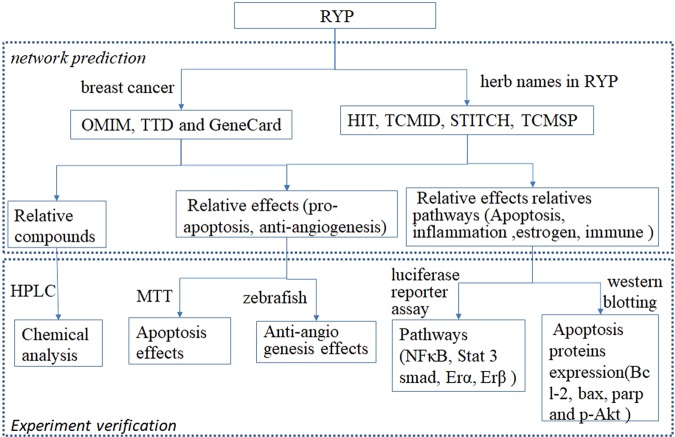
The flowchart of RYP application to breast cancer.

Therefore, in this paper, a network pharmacology approach was first employed to predict possible mechanism of RYP. Bioactive compounds, target and biological function enrichment for RYP were searched using network. Then experiments *in vivo* and *in vitro* were designed based on above results. Combining network approach and experiments (Fig. 1), the possible mechanism of RYP could be more easily focused than only by performing experiments without objectives.

## Results

### Results of network prediction

We searched the OMIM, TTD and GeneCard database using breast cancer as key word in order to find possible target gene/locus which might relate to breast cancer. Results showed about 345 genes might involve in breast cancer. Among them, the most relevant genes to breast cancer were estrogen, VEGF, Erb, Transforming Growth Factor Beta, Tumor Necrosis Factor, Interferon, AKT, SMAD Family Member, *et al*. (Table 1).

**Table 1:** Results of target gene for breast cancer.

Database	Gene/Locus name
OMIM	RAD54, S. cerevisiae, homolog-like
	Prostate and breast cancer overexpressed 1
	Estrogen receptor 1
	Breast cancer metastasis suppressor 1
	Breast cancer, type 3
	Breast cancer antiestrogen resistance 1
	Tumor protein p53
TTD	Receptor protein-tyrosine kinase erbB-4
	Transcription factor AP-1
	RAC-alpha serine/threonine kinase; AkT
	Estrogen receptor alpha
	VEGF-2 receptor
	Erbb2 tyrosine kinase receptor
	Estradiol 17 beta dehydrogenase 1
	Nuclear factor NF-kappa-B
	Estradiol 17 beta-dehydrogenase 1
	Estrogen receptor
	Receptor protein-tyrosine kinase erbB-2
	Estrogen receptor beta
GeneCard	Tumor Protein P53
	Erb-B2 Receptor Tyrosine Kinase 2
	Epidermal Growth Factor Receptor
	Estrogen Receptor 1
	AKT Serine/Threonine Kinase 1
	Epidermal Growth Factor
	BCL2 Associated X, Apoptosis Regulator
	Transforming Growth Factor Beta Receptor 2
	Estrogen Receptor 2
	Fibroblast Growth Factor Receptor 2
	Fibroblast Growth Factor Receptor 1
	Interleukin 6
	SMAD Family Member 4
	Vascular Endothelial Growth Factor A
	BCL2, Apoptosis Regulator
	Transforming Growth Factor Beta 1
	Fibroblast Growth Factor Receptor 4
	Signal Transducer And Activator Of Transcription 3
	Mitogen-Activated Protein Kinase 1
	Tumor Necrosis Factor
	Breast Cancer Anti-Estrogen Resistance 3
	Vascular Endothelial Growth Factor C
	Fibroblast Growth Factor 2
	BCL2 Like 1
	Erb-B2 Receptor Tyrosine Kinase 3
	Fibroblast Growth Factor Receptor 3
	Transforming Growth Factor Alpha
	Tumor Necrosis Factor Superfamily Member 10
	Interferon Gamma
	AKT Serine/Threonine Kinase 2
	Transforming Growth Factor Beta Receptor 1
	NFKB Inhibitor Alpha
	Interleukin 1 Beta
	SMAD Family Member 2
	Erb-B2 Receptor Tyrosine Kinase 4
	Signal Transducer And Activator Of Transcription 1

Next, we searched HIT, TCMID, STITCH, TCMSP database using herb names in RYP formula as key words in order to find possible relative genes, chemical compounds, reactions, pathways, biological processes, molecular dataset, diseases, and so on. Results showed as following:

About 78 genes might involve in therapeutic process for RYP formula application to breast cancer. Among them, the most relevant genes to RYP might be TNF, BCL2, AKT1, ICAM1, PARP1, ESR1, NFKBIA, ESR2, PPARA, PPARG, EGF, BCL2L1, EGFR, IL6, BAX, STAT3, TGFB1, VEGFA, *et al*.

About 47 chemical compounds in RYP formula might be bioactive ingredients (Table 2), including icariin, colchine and so on.

**Table 2:** Results of chemical compounds in RYP.

	ChemName		ChemName
1	beta-elemene	25	Stigmasterol
2	Ginkgetin	26	luteolin
3	beta-sitosterol	27	apigenin
4	poriferast-5-en-3beta-ol	28	icariin
5	13657-68-6	29	beta-elemene
6	Besigomsin	30	Hemo-sol
7	bilobetin	31	quercetin
8	pulegone	32	hyperin/hyperoside
9	oleanolic acid	33	CAM
10	beta-sitosterol	34	Vetol
11	hydroxytyrosol	35	colchine
12	baohuoside i	36	icariin
13	isoliquiritigenin	37	DFV
14	salidroside	38	Robinetin
15	Hirsutrin	39	Isoginkgetin
16	3,5,7-Trihydroxy-4′- methoxyl-8-prenylflavone -3-O-rhamnopyranoside	40	linolenic acid
17	tricin	41	NON
18	Germacron	42	Anhydroicaritin
19	emodin	43	lauric acid
20	oleanolic acid	44	TMH
21	Gastrodin	45	DOB
22	Terragon	46	Methyleugenol
23	Chryseriol	47	succinic acid
24	kaempferol		

**Figure 2:**
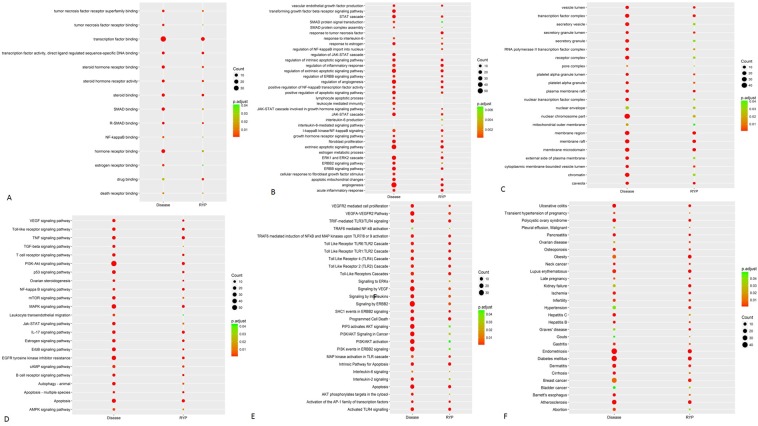
RYP’ Influence on enrichment of molecular functional (**A**), biological process (**B**), cellular components (**C**), KEGG signaling pathway (**D**), Reactome signaling pathway (**E**) and diseases (**F**).

About 265 kind molecular functions might relate to RYP. Among them, the significant molecular functions were listed as following: steroid hormone receptor activity, death receptor binding, tumor necrosis factor (TNF) receptor binding, steroid binding, transcription factor binding, drug binding, estrogen receptor binding, tumor necrosis factor(TNF) receptor superfamily binding, steroid hormone receptor binding, SMAD binding, NF-kappaB binding, hormone receptor binding, transcription factor activity, *et al*. (Fig. 2A).

RYP might involve in 4333 kind biological processes. Among them, the significant biological processes were listed as following: angiogenesis, toll-like receptor signaling pathway, leukocyte mediated immunity, transforming growth factor beta(TGFβ) receptor signaling pathway, SMAD protein complex assembly, estrogen metabolic process, apoptotic mitochondrial changes, vascular endothelial growth factor (VEFG) production, fibroblast migration, interleukin-6 (IL-6) production, response to tumor necrosis factor (TNF), ERBB signaling pathway, ERBB2 signaling pathway, tyrosine phosphorylation of Stat3 protein, response to estrogen, cellular response to fibroblast growth factor stimulus, positive regulation of NF-kappaB transcription factor activity, *et al*. (Fig. 2B).

RYP might involve in 108 kind cellular components. Among them, top 10 of cellular components were as following: chromatin, nuclear envelope, transcription factor complex, mitochondrial outer membrane, caveola, external side of plasma membrane, secretory granule, platelet alpha granule, platelet alpha granule lumen, vesicle lumen, *et al*. (Fig. 2C).

RYP might involve in 158 kind KEGG signaling pathways. Among them, the obvious KEGG signaling pathways were as following: ErbB signaling pathway, NF-kappa B signaling pathway, mTOR signaling pathway, PI3K-Akt signaling pathway, AMPK signaling pathway, apoptosis, TGF-beta signaling pathway, VEGF signaling pathway, Toll-like receptor signaling pathway, Jak-STAT signaling pathway, TNF signaling pathway, leukocyte trans-endothelial migration, ovarian steroidogenesis, estrogen signaling pathway, *et al*. (Fig. 2D).

RYP might involve in 399 kind Reactome signaling pathways. Among them, the obvious Reactome signaling pathways were as following: apoptosis, toll Like Receptor 4 (TLR4) Cascade, toll Like Receptor 2 (TLR2) cascade, signaling to ERKs, signaling by VEGF, PI3K/AKT activation, AKT phosphorylates targets in the cytosol, signaling by Interleukins, activation of the AP-1(Activator Protein-1) family of transcription factors, interleukin-2 signaling, signaling by ERBB2,PIP3 activates AKT signaling, PI3K events in ERBB2 signaling, VEGFA-VEGFR2 pathway, VEGFR2 mediated cell proliferation, *et al*. (Fig. 2E).

253 kind diseases might relate to RYP. Among them, the obvious diseases were respectively as following: malignant, endometriosis, gastritis, abortion, infertility, kidney failure, late pregnancy, ovarian disease, pancreatitis, polycystic ovary syndrome, bladder cancer, breast cancer, *et al*. (Fig. 2F).

To sum up, network results showed that RYP formula were related to breast cancer. Its possible mechanism might associate with apoptosis, inflammatory, estrogen, TGF-beta, VEGF, fibroblast proliferation, NF-kB, *et al*. Therefore, next experiments were designed according to these results.

### Chemical analysis results of RYP formula

**Figure 3:**
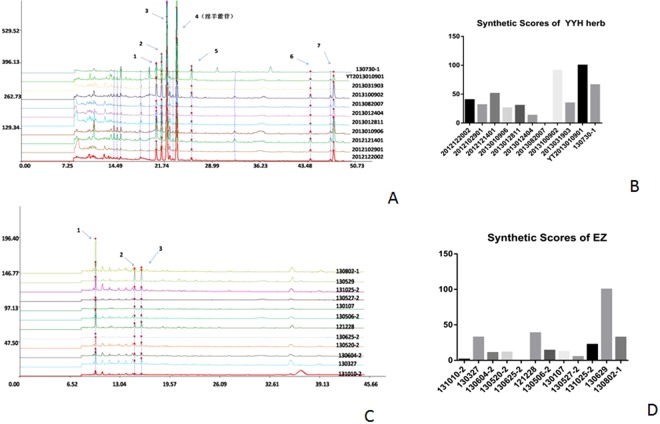
Chromatographic fingerprints and synthetic scores for HHY (**A**,**B**), EZ (**C**,**D**) at 270 nm.

**Figure 4:**
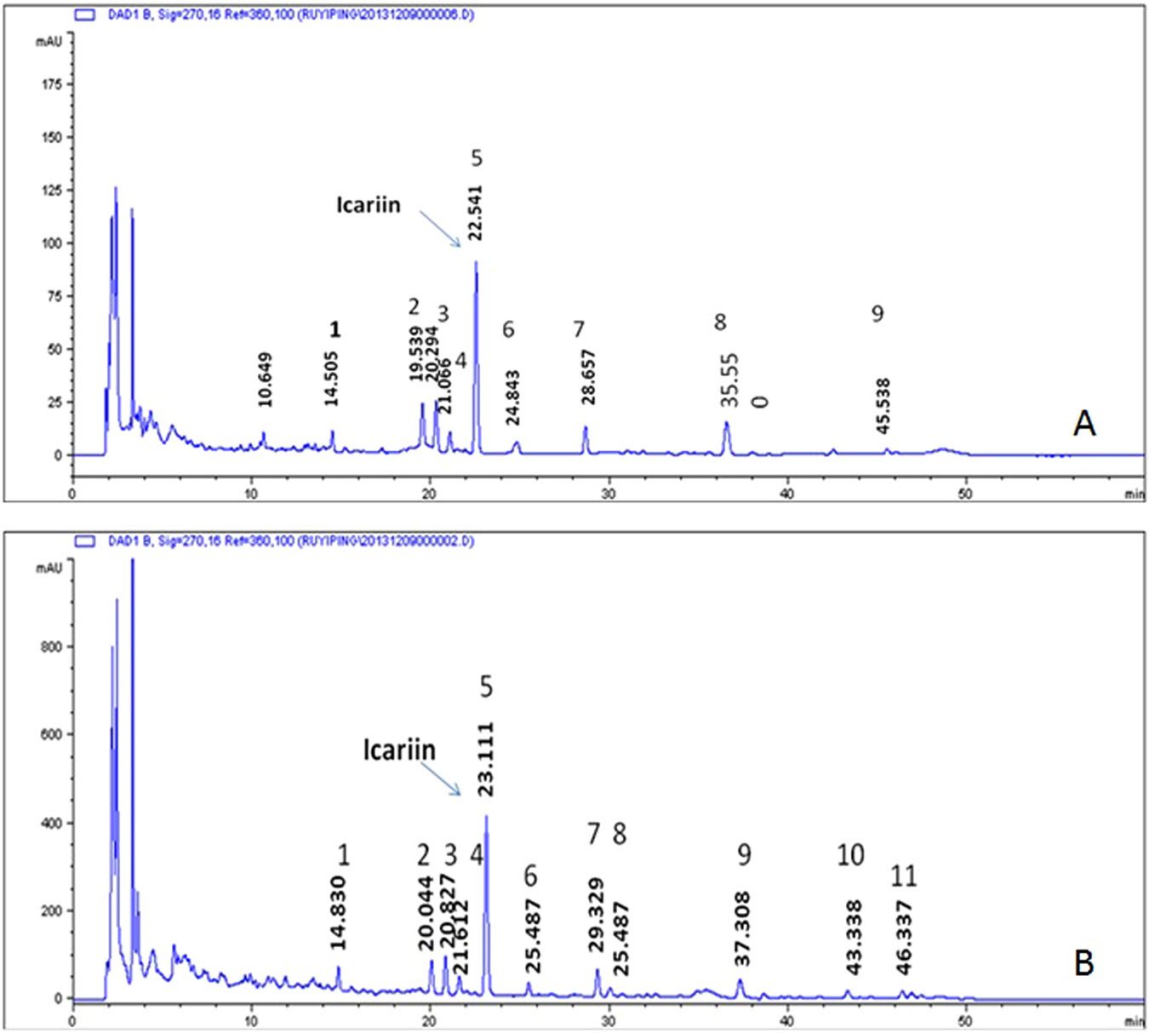
Chromatograms for RYP decoction (**A**) and dry extract (**B**) at 270 nm.

To analyze chemical ingredients in RYP and its single herbs, the HPLC fingerprints were obtained and analyzed different batches from different companies (Table 3). As showed in the Figs 3 and 4, main peaks were well separated. Under the wavelength of 270 nm, *Epimedium brevicornu Maxim* had seven common peaks (Fig. 3A), including icariin (Peak 4th) while *Rhizoma zedoariae* had three peaks (Fig. 3C). RYP Decoction had nine peaks (Fig. 4A) while dry extract had eleven peaks (Fig. 4B).

**Table 3:** Sources of Chinese herbal medicine.

Herbs	Batch No.	Company	Origin
YYH	2012122002	Shanghai DeHua Pharmacutical Corporation	LiaoNing
	2012102901	Shanghai DeHua Pharmacutical Corporation	LiaoNing
	2012121401	Shanghai HuaPu Pharmacutical Corporation	SiChuan
	2013010906	Shanghai HuaPu Pharmacutical Corporation	NeiMeng
	2013012811	Shanghai DeHua Pharmacutical Corporation	LiaoNing
	2013012404	Shanghai HuaPu Pharmacutical Corporation	SiChuan
	2013082007	Shanghai DeHua Pharmacutical Corporation	LiaoNing
	2013100902	Shanghai HuaPu Pharmacutical Corporation	LiaoNing
	2013031903	Shanghai HuaPu Pharmacutical Corporation	LiaoNing
	YT2013010901	Shanghai YuTianCheng Pharmacutical Corporation	GanSu
	130730-1	Shanghai Wan Shi Cheng Reed Sinopharm Products Ltd	LiaoNing
EZ	131010-2	Shanghai Wan Shi Cheng Reed Sinopharm Products Ltd	GuangXi
	130327	Shanghai YangHeTang Pharmacutical Corporation	GuangXi
	130604-2	Shanghai Wan Shi Cheng Reed Sinopharm Products Ltd	GuangXi
	130520-2	Shanghai Wan Shi Cheng Reed Sinopharm Products Ltd	GuangXi
	130625-2	Shanghai Wan Shi Cheng Reed Sinopharm Products Ltd	SiChuan
	121228	Shanghai Kangqiao Pharmacutical Corporation	ZheJiang
	130506-2	Shanghai Wan Shi Cheng Reed Sinopharm Products Ltd	YunNan
	130107	Shanghai Kangqiao Pharmacutical Corporation	ZheJiang
	130527-2	Shanghai Wan Shi Cheng Reed Sinopharm Products Ltd	GuangXi
	131025-2	Shanghai Wan Shi Cheng Reed Sinopharm Products Ltd	SiChuan
	130629	Shanghai Kangqiao Pharmacutical Corporation	ZheJiang
	130802-1	Shanghai Wan Shi Cheng Reed Sinopharm Products Ltd	YunNan

The established standard curve of icariin (y = 2443.4x + 18.28) exhibited a good linearity (correlation coefficient above 0.9999) within the range of 0.48 and 2.4 μg. Results (Table 4) showed RSDs (Relative Standard of Deviations) of the precision were not more than 4%. RSDs of stability (Table 5) and repeatability (Table 6) were also found less than 6%. These suggested the HPLC method was accurate, stable, repeatable and suitable for next analysis.

**Table 4:** Precision tests of HPLC.

Peak NO.	Peak area						
	S1	S2	S3	S4	S5	S6	*RSD* (%)
1	1216.6	1224.4	1306.4	1307.9	1318.9	1228.7	3.82
2	990.2	994.6	979.1	982.8	985.4	987.3	0.55
3	589.2	593.8	572	578.3	564.9	576	1.86
4	3346.9	3345.6	3350.1	3359.7	3353.2	3348.1	0.15
5	335.1	335.6	330.5	362.7	357.4	335.8	3.96
6	1213.7	1217	1221.7	1227	1227.7	1215.4	0.49
7	120.4	120.7	114.8	121.9	116	120.3	2.42

**Table 5:** Stability tests of HPLC.

Peak NO.	Peak area						
	S1	S2	S3	S4	S5	S6	*RSD* (%)
1	693.1	696.1	695.6	704.8	700.5	698.5	0.59
2	852.2	854.4	861.6	864.2	857.6	855.8	0.53
3	585.9	581.9	597.4	598.8	600.6	586.9	1.34
4	3373.4	3381.4	3359.4	3391.2	3354.5	3362.3	0.42
5	303	304.3	264.6	303.7	288.2	306.5	5.53
6	833.4	846.7	823.9	838.2	828.8	840.4	0.99
7	115.5	112.8	113.9	116.4	116.8	114.5	1.33

**Table 6:** The repeatability tests of HPLC.

Peak NO.	Peak area						
	S1	S2	S3	S4	S5	S6	*RSD* (%)
1	652	726.2	675.8	598.9	701.5	633.9	6.96
2	806.4	891.2	830.9	795.2	794.7	793.1	4.68
3	553.3	620.5	580.2	534.3	534.8	535.4	6.20
4	3221.4	3486.4	3281	3281.2	3291.7	3233.6	2.91
5	387.2	316.4	336	329.4	329.2	356.1	7.45
6	798.6	859.5	809.6	868.5	877.3	888.5	4.38
7	110.5	121.3	114	117	119.1	111.3	3.75

Using above method, the icariin contents in different batches of *Epimedium brevicornu Maxim* were determined. The result (Table 7) showed that contents of different batches were variable with RSD (36.20%). Further analysis results showed (Fig. 3B) that synthetic scores of different batches in 2012 were less than in 2013, indicating that the longer storage time, the less effective ingredients. Among them, sore of YT2013010901 was the highest, which could be used for the following experiment. Twelve batches of *Zingiberis Rhizoma* were respectively from GuangXi, Sichuan and Zhejiang (Table 3). Synthetic sore showed (Fig. 3D) that 130629 from Zhejiang was the best batch which could be used for further test. Furthermore, the content results of RYP showed (Table 8) that three batches of dry extract and decoction were consistent (RSD < 2%) and icariin content in dry extracts were ten times more than decoction.

**Table 7:** Contents of icariin in decoction for HHY.

Batch No.	Origin	Icariin (mg/g)
2012122002	LiaoNing	1.0342
2012102901	LiaoNing	0.9247
2012121401	SiChuan	1.1752
2013010906	NeiMeng	0.8577
2013012811	LiaoNing	0.9096
2013012404	SiChuan	0.6924
2013082007	LiaoNing	0.5238
2013100902	LiaoNing	1.6818
2013031903	LiaoNing	0.9642
YT2013010901	GanSu	1.7938
130730-1	LiaoNing	1.3638
mean		1.0837
RSD(%)		36.20

**Table 8:** Contents of icariin in dry extract and decoction of RYP.

Batch No.	Sample	Content (mg/g)	RSD (%)
20131209001	Dry extract	16.68	
20131209002	Dry extract	16.98	1.21
20131209003	Dry extract	17.08	
20131209004	Decoction	0.41	
20131209005	Decoction	0.41	0.60
20131209006	Decoction	0.41	

### Effects of RYP on MCF-7 cell lines

**Figure 5:**
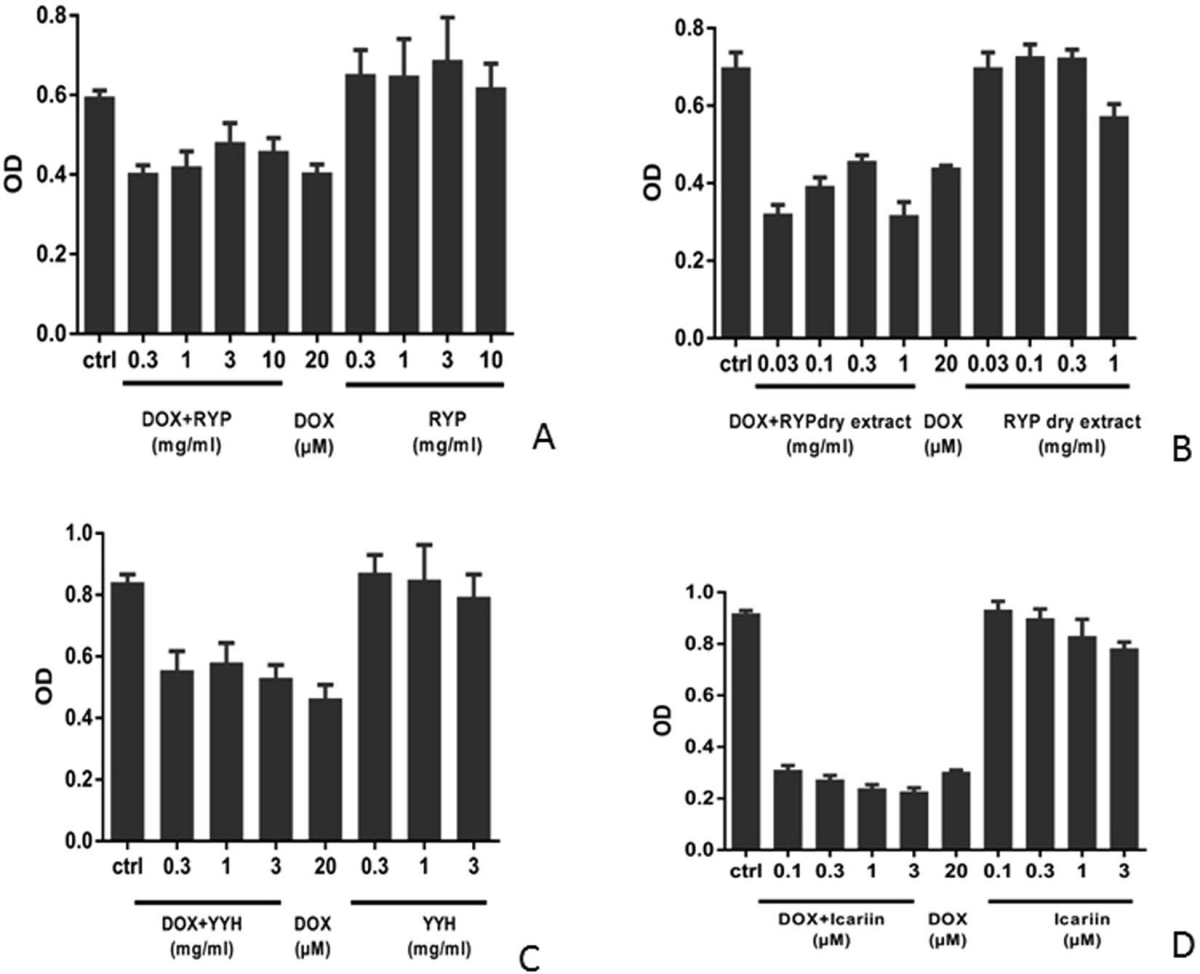
The effects of RYP decoction (**A**), RYP dry extract (**B**), YYH decoction (**C**) and icariin (**D**) combined with adriamycin on proliferation of MCF-7 cells (n = 3).

In order to observe effects of RYP on breast cancer cells, different concentrations of RYP decoction (Fig. 5A), dry extract (Fig. 5B), *Epimedium brevicornu Maxim* decoction (Fig. 5C) and icariin (Fig. 5D) were incubated with MCF-7 cells combined with or without adriamycin (DOX) for 24 h. Results showed (Fig. 5) cellular activities of DOX group were obviously lower than blank group. This indicated adriamycin could inhibit the proliferation of MCF-7 cells, suggesting that adriamycin as positive control was suitable. When incubated without adriamycin (DOX), anti-proliferation effects of drugs were not obvious. Only dry extract (Fig. 5B) and icariin (Fig. 5D) at high concentration exhibited anti-proliferation effects comparing with normal group. When incubated with adriamycin, not only dry extract (1 mg/ml) (Fig. 5B) expressed anti-proliferation effects, but also icariin (Fig. 5D) could inhibit proliferation of MCF-7 and expressed dose-dependence tendency. This suggested RYP dry extract could enhance effects of adriamycin and its possible ingredients might be icariin.

### Effects of RYP on zebrafish model

**Figure 6:**
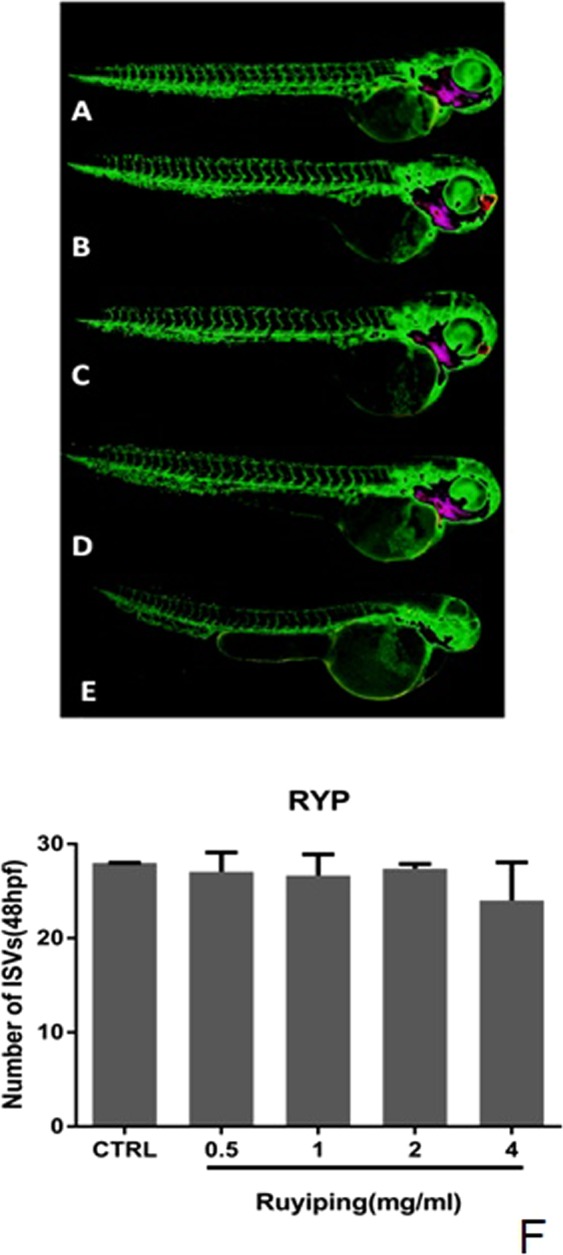
The effects of RYP on ISVs of Tg(fli1:EGFP). Embryos were treated without (**A**) or with RYP decoction at 0.5 mg/ml (**B**), 1 mg/ml (**C**), 2 mg/ml (**D**), 4 mg/ml (**E**) from 24 hpf to 48 hpf. Each group had six to eight items of zebrafish. Each test was done in triple. The numbers of ISVs were recorded at 24 h (**F**).

In order to observe anti-angiogenesis effects, low, middle and high concentrations of RYP decoction, single herb decoction and chemical pure compounds were respectively incubated with zebrafish for 24 hpf. Results showed that under the studied concentration range, single herb decoction and chemical pure compounds had no significant effects on zebrafish (data not listed). However, RYP decoction expressed weak anti-angiogenesis effects at high concentration (Fig. 6).

### Luciferase assay results of RYP and its components

**Figure 7:**
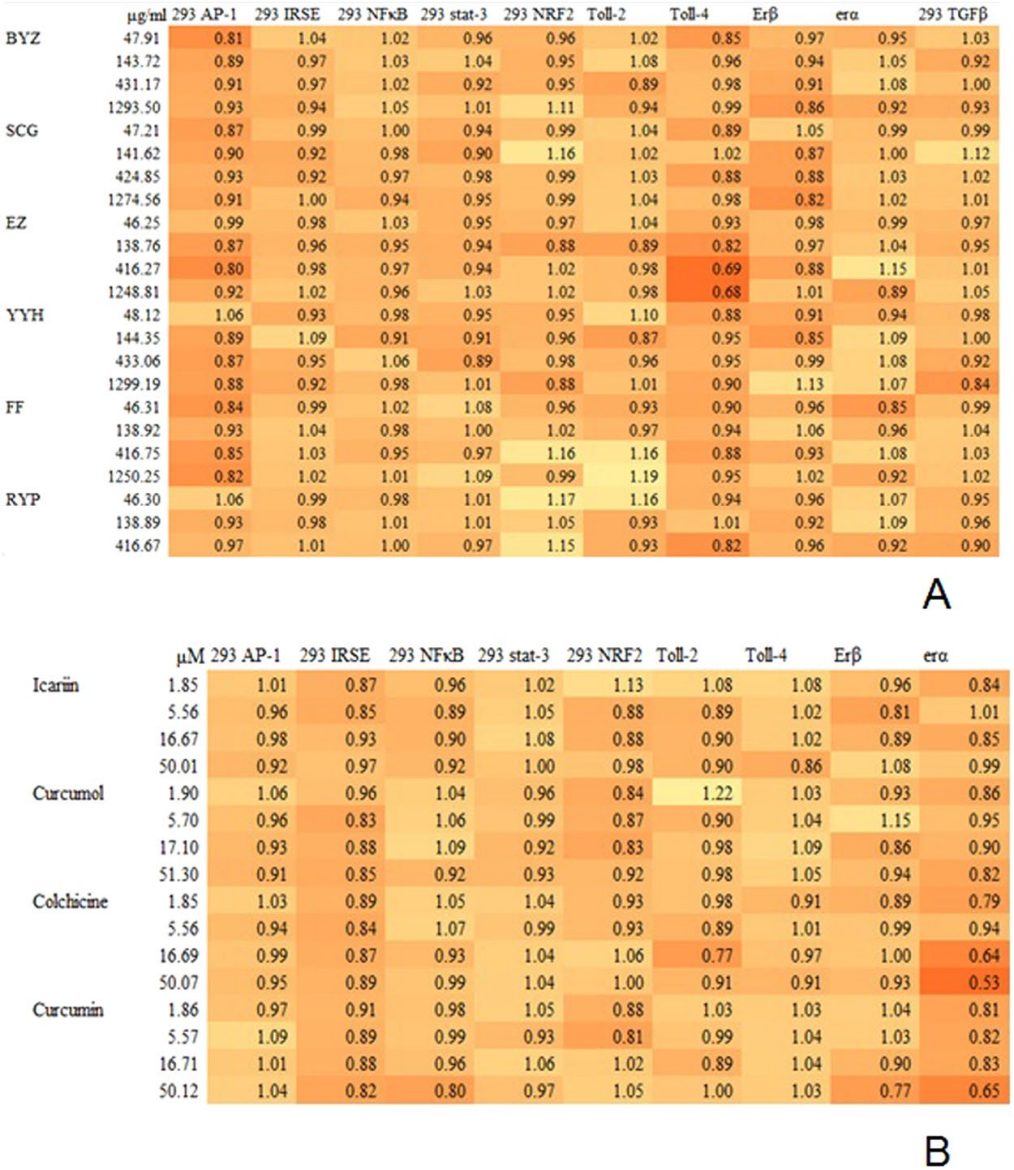
Effects of Herbs (**A**) and pure compounds (**B**) in RYP on growth of cell lines. The ratio of the average absorption value of drug group to blank group was calculated to evaluate toxicity of drugs on cell growth. Each data point was done in triplicate.

Before studying on bioactivity of drugs, we examined whether drugs had cytotoxicity on cell lines using methylene blue method. Data showed (Fig. 7) the ratios of the average absorption values of drugs groups to control group were between 80% and 120%, indicating that both herbs and pure compounds in RYP had no obvious toxicity on these cell lines.

Then the effects of drugs on transcriptional activities were investigated using luciferase reporter assay (Table 9). Results demonstrated that some drugs could influent some inflammation and estrogen relative pathways. For example, colchicine and curcumin could inhibit IRSE; RYP, FF, YYH, BYZ and curcumin could inhibit TGFβ; curcumin and YYH could inhibit Nuclear Factor κB (NFκB); RYP and BYZ could inhibit Erα; YYH, FF, RYP, curcumin could inhibit Erβ (Table 10).

**Table 9:** Luciferase assay.

Cell Line	Signaling pathway	Agonist	Agonist con (ng/mL)	Agonist Incubate time(h)
293 with AP-1		TPA	25	6
293 with GAS	stat 1	IFNγ		12
293 with ISRE	stat 1/2	Interferon α(IFNα)		6
293 with NFκB	NFκB	TNFα	25	6
293 with stat-3	Stat 3	Il-6	10	12
293 with TGFβ	Smad	TGFβ	2.5	6
293 with Erα		Erα		12
293 with Erβ		Erβ		12

ISRE: Interferon-Sensitive Response Element.

STAT-3:Signal transducer and activator of transcription 3.

TPA:12-O-tetradecanoylphorbol-13-acetate.

TNFα: Tumor Necrosis Factor α.

**Table 10:** Effects of pure compounds and herbs in RYP on luciferase activities.

Cell lines	Stimulator	Icariin	Curcumol	Colchicine	Curcumin	BYZ	SCG	EZ	YYH	FF	RYP
293 AP-1	TPA	(−)	(−)	(−)	(−)	(−)	(−)	(−)	(−)	(−)	(−)
293 IRSE	IFNα	(−)	(−)	↓	↓	(−)	(−)	(−)	(−)	(−)	(−)
293 NFκB	TNFα	(−)	(−)	(−)	↓	(−)	(−)	(−)	↓	(−)	(−)
293 stat-3	IL-6	(−)	↑	(−)	(−)	(−)	(−)	(−)	(−)	(−)	(−)
293 TGFβ	TGFβ	(−)	(−)	(−)	↓	↓	(−)	(−)	↓	↓	↓
Erα	Erα	(−)	(−)	↑	(−)	↓	(−)	(−)	(−)	(−)	↓
Erβ	Erβ	(−)	(−)	(−)	↓	(−)	(−)	(−)	↓	↓	↓

### Effects of RYP on protein expressions associated with apoptosis

**Figure 8:**
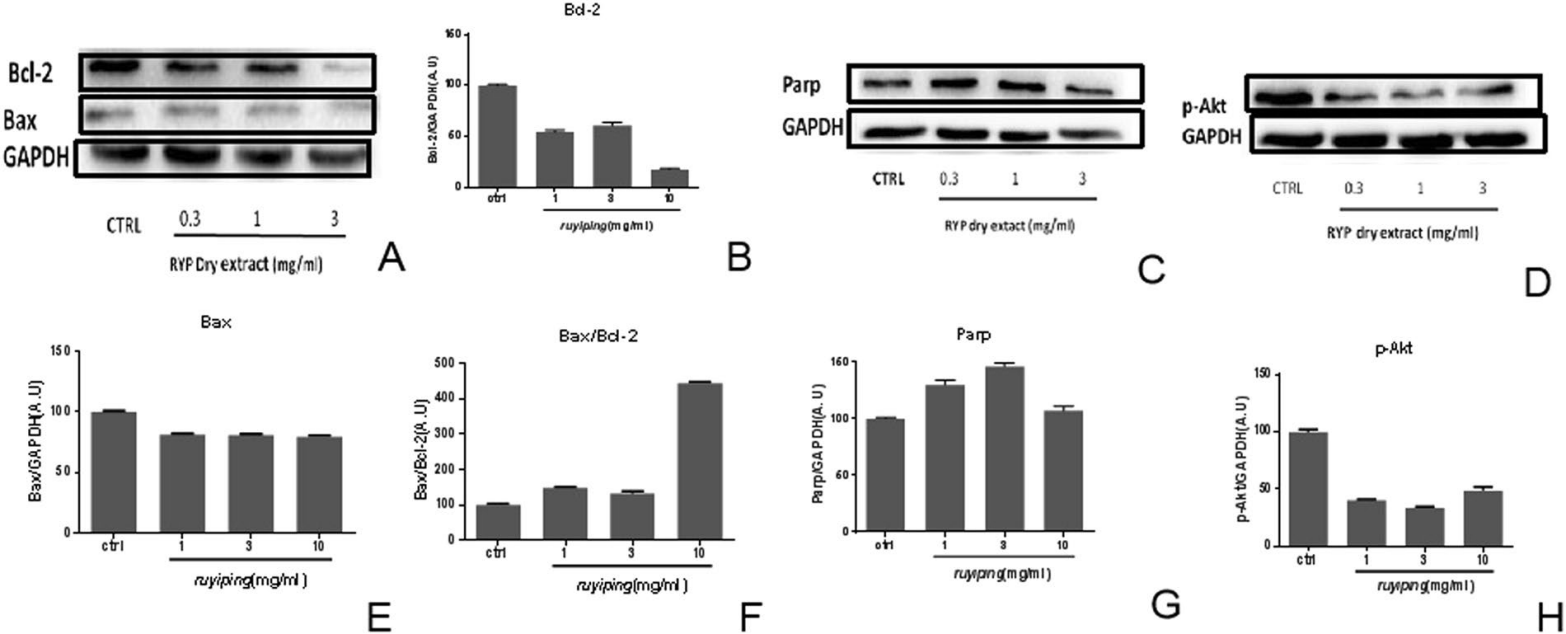
Effects of RYP dry extract on protein expression. MCF-7 cell lines were incubated without or with RYP dry extract at 1 mg/ml, 3 mg/ml,10 mg/ml for 24 h. The proteins of cells were extracted and determined by western blotting. The pictures of BCl-2 (**A**), bax (**A**), parp (**C**) and p-Akt (**D**) were taken. Then protein pictures were analyzed with software. The BCl-2 (**B**), Bax (**E**), parp (**G**) and p-Akt (**H**) protein expressions were obtained and ratios of Bax to BCl-2 were calculated (**F**). Each experiment was done in triple.

**Figure 9:**
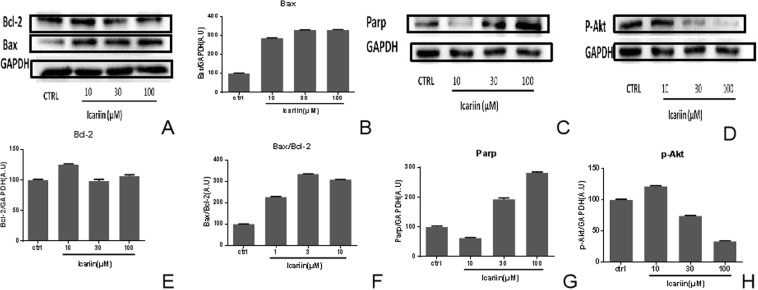
Effects of icariin on protein expression. MCF-7 cell lines were incubated without or with Icariin at 10 μM, 30 μM and 100 μM for 24 h. The proteins of cells were extracted and determined by western blotting. The pictures of BCl-2 (**A**), bax (**A**), parp (**C**) and p-Akt (**D**) were taken. Then protein pictures were analyzed with software. The Bax (**B**), p-Akt (**D**), BCl-2 (**E**), parp (**F**) and p-Akt (**H**) protein expressions were obtained and ratios of Bax to BCl-2 were calculated (**F**). Each experiment was done in triple. (The KDA of Parp is about 116 and the KDA of p-akt is about 60. After blocking, the membrane was cut on 70 kDa, actually these two proteins come from one gel, Therefore, the control in (**C**,**D**) was same.

In order to explore the possible mechanism for breast cancer, effects of RYP formula on apoptosis relative proteins were observed using western blotting method. Different concentrations of RYP dry extract and its mark ingredient-icarrin were incubated with breast cancer cell lines-MCF7 for 24 h. Then proteins in cells were extracted and expressions were determined. Results showed that RYP dry extract could down-regulate BCl-2 (Fig. 8A,B) and p-Akt (Fig. 8D,H); up-regulate Bax/BCl-2 (Fig. 8F) and Parp (Fig. 8C,G); Icariin could down-regulate BCl-2 (Fig. 9A,E) and p-Akt (Fig. 9D,H), up-regulate Bax/BCl-2 (Fig. 9F) and Parp (Fig. 9C,G).

## Discussion

Network pharmacology is based on the analysis of network models and systems biology. Taking advantage of advancements in systems biology, a high degree of integration data analysis strategy and interpretable visualization provides deeper insights into the underlying mechanisms of TCM theories, including the principles of herb combination, biological foundations of herb or herbal formulae action and molecular basis of TCM syndromes. The network pharmacology research process usually begins from the identification of drug- or disease-related biological entities (gene, protein, and metabolite) and then proceeds by constructing drug- or disease-related networks that could reveal underlying relationships by analyzing network topology properties. Some researcher has tried applied systems pharmacology to study molecular targets and associated potential pathways of Danlu capsules in hyperplasia of mammary glands^17^. They have also identified the active compounds and significant pathways of Yinchenhao decoction^18^. These attempts give us a good inspiration. Thus our team has also tried to search some databases and use computational tools^19^, which were successfully applied to predict p-Glycoprotein inhibitors^20^, discovery core effective formulae in TCM^21^, mine the tumor clinical data of TCM^22^, identify serum lipid alteration^23^ and so on. These experiences provide a basis for us to study RYP formula. As a sophisticated formula in our hospital, clinical data of RYP have already confirmed that it is effective for relapse and metastasis of breast cancer^4^. But its mechanism is still not very clear and the herbs in the formulae are not very classical. These bring us difficulties to figure out its mechanism. Therefore, in this article, in order to screen possible target genes, compounds and pathways of RYP, network pharmacological prediction was performed using databases including OMIM, TTD, GeneCard, HIT, TCMID, STITCH, TCMSP, *et al*.

Our network results in this manuscript showed many pathways might involve in the therapeutic process for RYP formula application to breast cancer, including apoptosis, toll Like Receptor 4 (TLR4) Cascade, toll Like Receptor 2 (TLR2) cascade, signaling to ERKs, signaling by VEGF, PI3K/AKT activation, AKT phosphorylates targets in the cytosol, signaling by Interleukins, activation of the AP-1 family of transcription factors, interleukin-2 signaling, signaling by ERBB2, PIP3 activates AKT signaling, PI3K events in ERBB2 signaling, VEGFA-VEGFR2 pathway, VEGFR2 mediated cell proliferation, *et al*. Accordingly, some target genes may participate in these procedure, such as TNF, BCL2, AKT1, ICAM1, PARP1, ESR1, NFKBIA, ESR2, PPARA, PPARG, EGF, BCL2L1, EGFR, IL6, BAX, STAT3, TGFB1, VEGFA, and so on. Diseases associated with RYP formula may be endometriosis, obesity, breast cancer, ovarian disease, and so on. Potential active compounds might be icariin, emodin, colchine, *et al*. Although through network analysis, some potential mechanisms can be predicted, these results still required to be confirmed by experiments. Thus, several experimental techniques and models, including zebrafish model, MTT assays, luciferase assays and western blotting were performed as flowchart (Fig. 1).

First of all, quality control of formula was the most important. So HPLC method was performed to determine mark ingredients in formula. The results showed that the samples for next pharmacological experiments were controllable and consistent, guaranteeing following experimental repeatable.

Then in reference to pharmacological functions, as we known, TCM theory for establishing RYP formula was to remove toxin and dissipate nodule. “Remove toxin” might be relative to inflammatory and apoptosis pathways while “dissipate nodule” could involve in angiogenesis and fiber-genesis^24^. Breast cancer also connected with estrogen receptor^25,26^. Our primary results predicted by network were consistent with these literature in some degree. Therefore, we designed experiments to focus on the pathways associated with apoptosis, inflammatory, angiogenesis, estrogen, immune system, *et al*.

As for apoptosis, MTT test is a routine classic method to observe pro-apoptosis functions of drugs. MCF-7 cell lines belonged to human breast cancer cells and exhibited characteristics of mammary’ epithelial cells, so this cell lines were selected for MTT test. Data of MTT showed that dry extract of RYP and icariin could play synergetic pro-apoptotic effects on breast cancer cells combined with adriamycin, and icarrin might be an effective ingredient in RYP decoction. However, RYP decoction did not show obvious effects. To analyze reasons, dry extract of RYP was condensed from RYP’s decoction, effective ingredients were more than decoction (Table 8).

As for angiogenesis, we all know that angiogenesis was crucial during tumor’s occurrence and development^27^. Anti-angiogenesis became a research focus for tumor so a high screening model-Tg (fli1a-EGFP) zebrafish were introduced to observe effects of RYP formula and its ingredients on angiogenesis^28–31^. Results of zebrafish showed that RYP, herbs and ingredients had no obvious angiogenesis effects. However, dry extract of RYP at high dose showed anti-angiogenesis trend.

As for signaling pathways, luciferase reporter assay, a high- through-out model, was applied to observe effects of RYP and its ingredients on transcriptions. Bioluminescence assay systems are being used increasingly in biology and medical research laboratories in addition to (or as alternatives to) fluorescence and chemiluminescence detection strategies. Luciferase enzymes isolated from different animal species have inherent variability in light emission, allowing two or more luciferase enzymes to be used in combination for multiplex analyses, including *in vivo* imaging, cell viability and single and dual-spectral luciferase reporter assays. Additionally, luciferase reactions are classified as having either flash or glow kinetics, which have specific detection sensitivities and emission duration times to accommodate different experimental designs^32^. Results of luciferase assay in this demonstrated that some ingredients in RYP formula did have bioactivities on Smad, NFκB, IFN, erα and erβ pathways (Table 10). However, although our network data showed that some factors such AP-1 family of transcription, and TLR signaling pathway might participate in the procedure of RYP, our experiments did not verify them.

In order to figure out mechanism of RYP pro-apoptotic effects, expressions of apoptotic proteins such as Bax, BCl-2, PARP and Akt were determined. For Bcl2-and Bax, Bcl-2 expressions decreased with increasing of RYP dry extract, while Bax expressions had no obvious change. Meanwhile, icarrin, the main ingredient of RYP, could increase Bax expression and decrease Bcl-2. For PARP, RYP dry extract had no significant effects while icarrin could elevate PARP expression. For Akt, RYP dry extract had no significant effects while icarrin could decrease Akt expression. These results indicated that the pro-apoptotic effects of RYP might relate to the proteins including Bcl2. And the possible effective ingredients might be icarrin.

In summary, the quality of RYP formula was controllable and icariin could be selected as mark ingredient; RYP expressed anti-breast tumor effects, which could be associated with inhibiting expression of TGFβ, promoting cells apoptosis and anti-angiogenesis. Furthermore, above results were consistent with primary network pharmacology-based prediction in some degree, but not all the results predicted by network can be verified by experiments. Network can help us narrow areas, focus on crucial factors, save money as well as time, but the predicted results are required to be confirmed by further experiments.

## Methods

### Network Prediction

**Figure 10:**
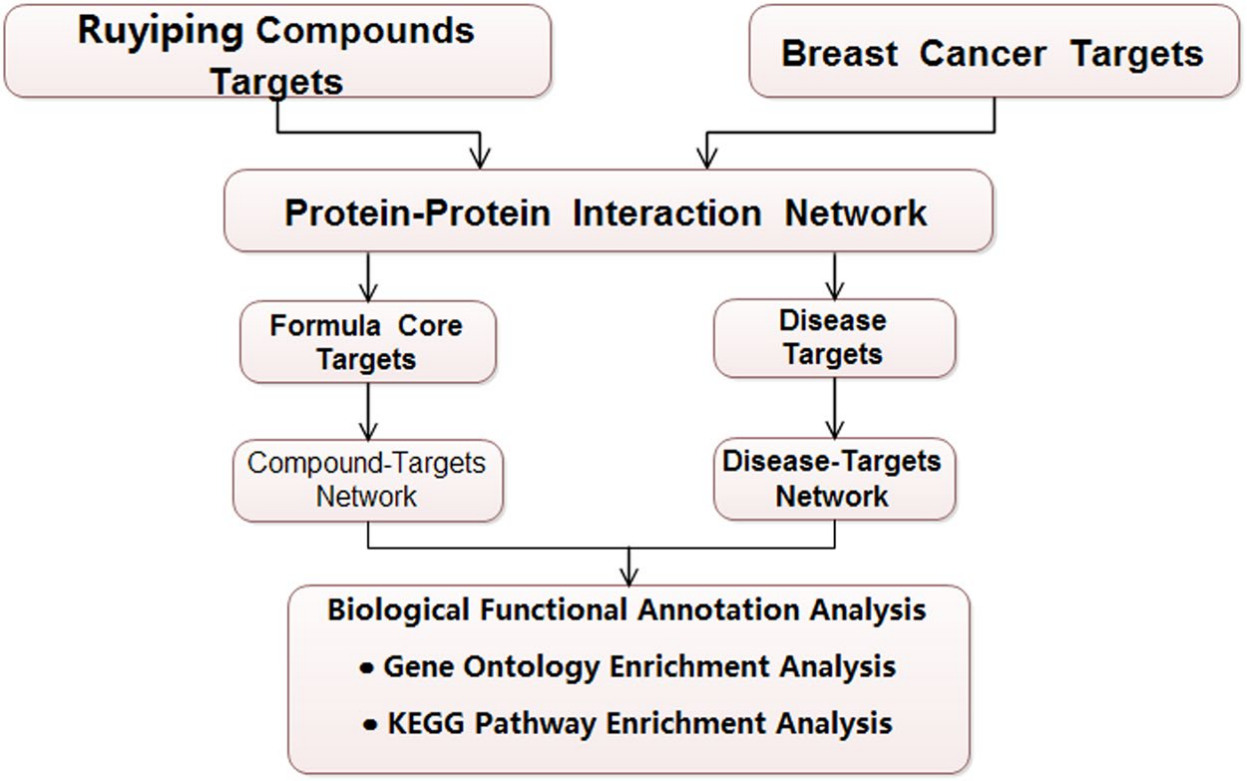
The workflow of network prediction.

The principal idea about targets selection is: each ingredient in TCM formula such as RYP may impact on multiple target genes while each target gene may have interactions with multiple compounds^14^. Therefore, before experiment network prediction was performed according to the following workflow (Fig. 10) and the equation. When P is less than 0.05, the probability of event that the target has interaction with the current ingredient is rare.

$$P(X\ge k)={\sum _{m=0}^{k}{C}_{n}^{m}(\frac{g}{n})}^{m}{(1-\frac{g}{n})}^{n-m}$$

n:the total number of ingredients; g: the average number of ingredients with which each target gene has interaction; k: the number of ingredients with which current target gene has interaction.

### Target gene analysis using breast cancer as key word

Prediction of possible Gene/Locus for breast cancer was carried out by searching OMIM, TTD and GeneCard Database using breast cancer as key word. Here, OMIM is a database of comprehensive, authoritative compendium of human genes and genetic phenotypes, which normally is used for Disease-gene retrieval. Its webpage is “http://www.omim.org/”^33^. TTD is a therapeutic target database which can provide information about the known and exploring therapeutic protein and nucleic acid targets, the targeted disease, pathway information, and the corresponding drugs. Its webpage is http://bidd.nus.edu.sg/group/cjttd/ ^34^. GeneCard is a human gene database. It is a searchable, integrative database that provides comprehensive, user-friendly information on all annotated and predicted human genes. It automatically integrates gene-centric data from ~125 web sources, including genomic, transcriptomic, proteomic, genetic, clinical and functional information.

### Target gene and bioactive compounds analysis using RYP and its herbs as key words

Prediction of possible target gene and chemical compounds for RYP was performed by searching HIT, TCMID, STITCH, TCMSP Database. Here, HIT is a comprehensive and fully curative database for linking herbal active ingredients to targets. It can be used for herbal ingredients’ targets identification. Its webpage is http://lifecenter.sgst.cn/hit/^35^. TCMID is a Traditional Chinese medicine integrated database. It is a comprehensive database to provide information on drug-herb and its ingredient, prescription, target, and disease, so it can be used for comprehensive analysis for TCM biological sciences. Its webpage is http://www.megabionet.org/tcmid/^36^. STITCH is a chemical-protein interactions database which can provide known and predicted interactions of chemicals and proteins. It can be sued for chemical-protein interaction retrieval. Its webpage is http://stitch.embl.de/^37^. TCMSP is a Traditional Chinese medicine system, pharmacology database and analysis platform which can provide information on relationships between drugs, targets, and diseases. It can be used for comprehensive analysis for TCM biological sciences. Its webpage is http://tcmspnw.com^38^.

Key words were RYP herbs including *Epimedium brevicornu Maxim* (YYH), *Rhizoma zedoariae* (EZ), *Iphigenia* (SCG), *Nidus Vespae* (FF) and *Holboellia fargesii Reaub* (BYZ). Relevance Threshold value of Gene target was 400. Threshold value of chemical compound was 0.1. Significant Threshold value of Gene target: 0.05.

### Biological function and disease enrichment analysis using RYP and its herbs as key words

Gene Ontology (GO) biological processes, KEGG pathway profiling and Reactome pathway profiling were used for functional enrichment analysis of the sets of selected target proteins and of disease related genes. Here, GO can be classified into three categories: molecular Function, biological process and cellular component. KEGG pathway maps are biological interpretaion of higher-level systemic functions. KEGG pathway mapping is the process to map molecular datasets, especially large-scale datasets in genomics, transcriptomics, proteomics, and metabolomics. Reactome is a database of reactions, pathways and biological processes.

In this article, Gene Ontology (GO), KEGG and Reactome were applied for RYP analysis. The functional enrichment tool DAVID23 was used to calculate GO enrichment, KEGG and Reactome pathway enrichment. Only KEGG pathway and Reactome pathway with P-values < 0.05 were included (corrected using the Benjamini method). The relationship similarity and AUC (Area under curves) for KEGG and Reactome pathway were obtained. Disease ontology enrichment analysis was conducted according to a previous method. Diseases with a P-value < 0.05 (adjusted by the Bonferroni method) were included^39^.

## Experiment

### Chemicals and reagents

Pure chemical standards including icariin were bought from the Institute for the Control of Pharmaceutical and Biological Products of China (Beijing, China). HPLC grade methanol and acetonitrile (Merck, Darmstadt, Germany) were used as mobile phase while Dimethyl sulfoxide (DMSO) (Sangon biological company, Lot: SJ0509S4512J) was selected as co-solvent. Deionized water was made by a Milli-Q water system (Millipore, Bedford, MA, USA). VEGF Receptor tyrosine kinase Inhibitor (VRI) came from Calbiochem (Cat. No. 676481 MA, USA).

### Plant materials

Different batches of herbs in RYP formula, which were bought from different companies (Table 3), were stored in a well-ventilated room at 25 °C under the absence of light. All of them were identified by experienced pharmacist (Shi, Xiu Feng). About 10 g of herb sample was boiled with ten folds of water for 20 min (100 °C), then filtrated, mixed and concentrated to decoction sample (1 g crude powder/ml) for next experiment.

### Study on chemical ingredients of RYP formula

Pure chemicals were accurately weighed and dissolved in methanol to make into standard stock solutions, which were diluted into a series of concentrations for calibration curves. 1 ml of herb decoction was accurately pipeted, diluted with 10 ml methanol, extracted for 20 min under ultrasound, replenished with methanol to 10 ml, centrifuged and filtered through a 0.22 μm microporous membrane (Millipore Corporation, Billerica, MA, USA) for HPLC analysis.

Chemical analysis was carried out using an Agilent HPLC 1100 series system (Agilent Corporation, Waldbronn, Germany), including a low-pressure quaternary gradient pump, an auto-sampler, a column temperature controller, an online degasser, and a G1315B DAD. A Kromasil C_18_ column (5 μm, 250 mm × 4.6 mm, Cat. No. EH04704) was selected for analysis. Mobile phase consisted of solvent A (HPLC water) and solvent B (acetonitrile) with 1.0 ml/min flow rate at 25 °C.The gradient elution condition was following: 15 ~ 20% B at 0 ~ 10 min; 25 ~ 40% B at 10 ~ 45 min; 40 ~ 15% B at 45 ~ 55 min; 15% B at 55 ~ 60 min. The injection volume was 10 μl and wavelength was set at 270 nm.

To evaluate the precision, the same sample was injected in six times. To study the repeatability, the same batch herbs were processed into six samples at same method. To observe the stability of sample, an aliquot was injected respectively at 0, 3, 6, 9, 12 and 24 h.

In this study, HPLC fingerprints were evaluated with synthetic method which integrated both the AHP and CRITIC methods according to our previous literature^40^. The parameters were classified into three layers: the first layer indicated quantitative mark ingredient while the second and third layer respectively meant common and uncommon peaks. Weights of variable parameters were obtained according to the hierarchical system. Then the scores of different batches were calculated and the best batch of herb was selected for further pharmacological research.

### Effects of RYP formula on breast cancer cell lines

MCF-7 cell lines were obtained from the Shanghai Institute of Cell Biology, Chinese Academy of Sciences (Shanghai, China), were respectively cultured in DMEM medium containing 10% heat-inactivated fetal bovine serum, penicillin (100 U/ml) and streptomycin (100 mg/ml) at 37 °C under an atmosphere of 95% air and 5% CO_2_. Cells were routinely subcultured every 2 to 3 days and cells samples used were all in the logarithmic growth phase. Cell lines were cultured until 80% flask bottom covered and dissociated to single cell by typsin (0.25% EDTA).

Then MCF-7 Cell lines (1 × 10^4^cells/well) were respectively seeded into 96-well plates in culture medium over night and treated with various concentrations of samples at the indicated times (24 h). Volume per well was 200 μl. For each kind drug, five wells were repeated. Next, 20 µl of MTT reagent solution (5 mg/ml) was added to each well and the cells were incubated for 4 h at 37 °C. After the medium and MTT were removed, 150 µl DMSO was added to each well and placed on a plated shaker for 5 min at room temperature in order to dissolve water-insoluble formazan. Then the optical density (OD) was measured at 490 nm using a micro-plate reader (Bio-Rad, Hercules, California, USA). The inhibition rate (IR) was calculated as the following formula: percentage of inhibition = [1−(mean OD of experimental sample/mean OD of the control group)] × 100%. All of experiments were performed in triple.

### Effects of RYP formula on zebrafish model

All animal protocols were approved by the Animal Experimentation Ethics Committee of Shanghai University of TCM and meet the requirements of the ethical guidelines of Institute of Chinese Medical Sciences.

The transgenic zebrafish lines with Tg (fli-1:EGFP) were cultured according to zebrafish handbook^41^. Healthy transparent and regular embryos were picked out at the 1–4 cell stage. At 24 hpf, the healthy embryos were decorticated and distributed into a 24-well micro-plate with around 6~8 fish in each well. Then zebrafish were incubated with fresh media containing different drugs at low, middle and high concentrations for 24 h to assess anti-angiogenesis activity. The drugs included single herb decoction samples [*Epimedium brevicornu Maxim* (0.25, 0.5, 1 mg/ml), *Rhizoma zedoariae* (1, 2, 4 mg/ml), *Iphigenia* (0.5, 1, 2 mg/ml), *Nidus Vespae* (0.5, 1, 2 mg/ml) and *Holboellia fargesii Reaub* (0.5, 1, 2 mg/ml)], RYP decoction (0.5, 1,2, 4 mg/ml) and chemical pure compounds [curcumol (10, 30, 100 μM), Icariin (10, 30, 100 μM), colchicines (10, 30, 100 μM), curcumin (1, 3, 10 μM) and curcudione(10, 30, 100 μM)]. The embryos with fresh media were as blank control.

After 24hpf, the morphological transformation of embryos was observed with an Olympus Spinning Disk Confocal Microscope System (IX81 Motorized Inverted Microscope). Intersegmental vessels (ISVs) refer to the vessels which come from dorsal aorta (DA) or (posterior cardinal vein (PCV)) to Dorsal Longitudinal Anastomotic Vessels (DLAVs). If ISVs were elongated from DA or PCV and connected with DLAVs, these ISVs were considered as intact. Otherwise, they were considered as defective. The effects of drugs were evaluated by counting the numbers of defective and intact ISVs in each embryo. Then the index was calculated according to the equation: Angiogenesis index = number of intact ISVs + 0.5 × number of defective ISVs.

### Luciferase assay of RYP and its ingredients

HEK (Human Embryonic Kidney) 293 cells were obtained from the American Type Culture Collection (Manassas, VA). HepG2 cells were maintained in RPMI1640 medium (Gibco) supplemented with 10% heat-inactivated Fetal Bovine Serum (FBS, Gibco). HepG2 or HEK-293 cell lines stably harboring different response elements in pGL4.0 luciferase vector (Promega) were used for the signaling pathway reporter assay (Table 9).

HEK-293 cells were maintained in Dulbecco’s Modified Eagle Medium (DMEM, Gibco) containing 4.5 g/L glucose supplemented with 1% penicillin-streptomycin and 10% FBS. All cell lines were maintained in a humidified incubator with an atmosphere of 95% air and 5% CO_2_ at 37 °C or in a 37 °C hypoxic chamber that was perfused with a gas mixture of 1% O_2_, 5% CO_2_, and 95% N_2_.

Ten thousands of cells/well were plated in 96-well plates. After overnight incubation, cells were treated with different drugs at low, medium and high concentrations for 72 h. Each data point was done in triplicate. The experiment was in cells were fixed and stained with 0.5% methylene blue (sigma) in 50% ethanol for 2 h at room temperature, followed by washing with tap water to remove excess color. Plates were dried and then re-suspended in 1% lauroylsarkosine (sigma) and incubate for 3 h at room temperature. Cell growth was quantified based on the amount of methylene blue absorbed into cellular proteins measured by spectrophotometer (Molecular Devices) at 595 nm. The ratio of the average absorption value of drug group to blank group was calculated to evaluate toxicity of drugs on cell growth. Each data point was done in triplicate.

Cells lines with reporters were plated at 2 × 10^4^ cells/well in 96-well plates. After confluence, cells were treated with different pure compounds and drugs at low, medium and high concentrations. Then cells were incubated with corresponding agonists for 6 h or overnight (12 h) and lysed using 40 μl luciferase lysis buffer (25 mM Tris-HCl, 2 mM DTT, 2 mM CDTA, 10% glycerol,1% Triton X-100) for 10 min. 20 μl of lysed samples were transferred into 96 well white plate (Corning Costar® plate) for luminescent assays. 30 μl luciferase assay buffer (20 mM Tris-HCl, 1 mM NaHCO_3_, 2.5 mM MgSO_4_, 0.1 mM EDTA, 10 mM DTT, 60 μM coenzyme-A lithium, 225 μM potassium luciferin, 250 μM ATP) were added and transcriptional activity was determined by measuring the activity of firefly luciferase in a multi-well plate luminometer (Tecan, Durham, NC) using luciferase reporter assay (Promega) according to the manufacturer’s instructions. All firefly luciferase values were normalized to renilla luciferase in order to compare the transfection efficiencies. Inducing fold >2 or inhibiting rate >50% was considered as significance. IC_50_ was defined as the concentration of drug that inhibited stimulator-triggered luciferase reporter activation by 50%.

### Effects of RYP and its ingredients on expressions of proteins

MCF-7 cells in the logarithmic growth phase were seeded in 35 mm culture dishes at a density of 3 × 10^5^ cells/ml and incubated for 24 h. Subsequently, the adherent cells were respectively incubated with RYP dry extract at 1 mg/ml, 3 mg/ml,10 mg/ml or icariin at 10 μM, 30 μM and 100 μM for 24 h, while the control groups were treated with culture medium containing 10% FBS.

The adherent cells were washed twice with ice-cold phosphate-buffered saline (PBS), lysed with RIPA buffer (RIPA buffer/protease inhibitors/EDTA = 100:1:1; Thermo Beyotime, China) for 5~10 min on ice, harvested with a scraper, and centrifuged for 15 min at 14,000 g under 4 °C. The resulting supernatants containing the protein fraction were stored at −80 °C until analysis.

The protein concentrations were determined with a Bicinchoninic Acid (BCA) Protein Assay Kit (Pierce, USA) according to the manufacturer’s instructions. Afterwards, all protein samples were mixed with Laemmli loading buffer (Beyotime Institute of Biotechnology, China), boiled for 3~5 min, and stored at −20 °C until analysis.

Equivalent amounts of proteins were subjected to 8% sodium dodecyl sulfate-polyacrylamide gel electrophoresis and transferred to polyvinyl lidene difluoride membranes (Beyotime Institute of Biotechnology, China). Subsequently, the membranes were blocked with 5% skim milk (Beyotime Institute of Biotechnology) and shaken at 80r/min for 1 h under room temperature on a shaker and incubated with specific primary antibodies (1:1000 dilution in PBS) against β-actin, Bcl-2, Bax, PARP, p-Akt (Santa Cruz Biotechnology Inc., USA) overnight at 4 °C. The membranes were washed three times with western cleaning solution (Beyotime Institute of Biotechnology, China), incubated with a horseradish peroxidase-conjugated secondary antibody (1:15,000 dilution times in ultrapure water) for 30 min at room temperature in a dark place, and washed three times with western cleaning solution. Finally, immune-reactive proteins were detected by enhanced chemo-luminescence (Odyssey Infrared Imaging System, LI-COR Co. LTD, USA). In this experiment, β-actin was used as an internal control to confirm that the amounts of loaded protein were equal. All experiments were repeated at least three times.

### Statistical analysis

All tests were carried out at least in triple with the values expressed as mean ± SD. The lab results were analyzed using One-way ANOVA (Analysis of variance) with *P* value < 0.05 as statistically significance.

## Supplementary information

Supplementary Information
