# Supplementary material for: Effects and possible mechanism of Ruyiping formula application to breast cancer based on network prediction
Source: Sci Rep. 2019 Mar 27;9:5249. doi: 10.1038/s41598-019-41243-9 (PMC6437177; doi:10.1038/s41598-019-41243-9)
Supplement: Supplementary file 1 — Supplementary Information [file 41598_2019_41243_MOESM1_ESM.pdf]

Effects and possible mechanism of Ruyiping formula application to breast cancer based on network prediction

Rui-Fang Xie[1], Sheng Liu[1], Ming Yang [1], Jia-Qi Xu[1], Zhi-Cheng Li[2], Xin Zhou[1*]

[1]Longhua Hospital affiliated to Shanghai University of Traditional Chinese Medicine, Shanghai, 200032, China.[2]Surgery,Shanghai Pu Dong Hospital, Shanghai, China.

Correspondences and requests for materials should be addressed to X.Z. (E-mail: 2479707904@qq.com)

R-F X and S. L. are equally contributed to the paper

BCL-2

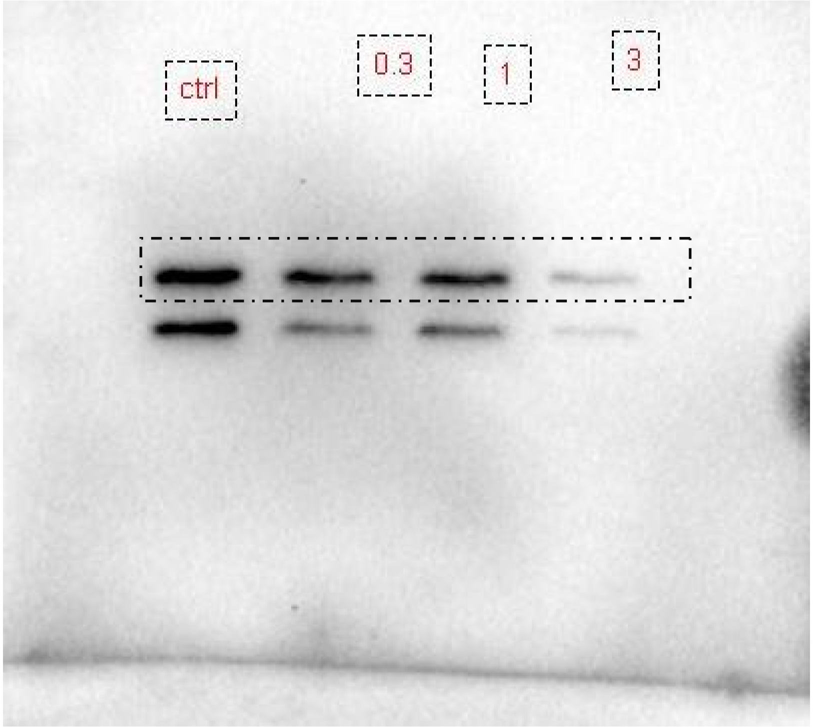

Effects of RYP dry extract on BCL-2

BAX

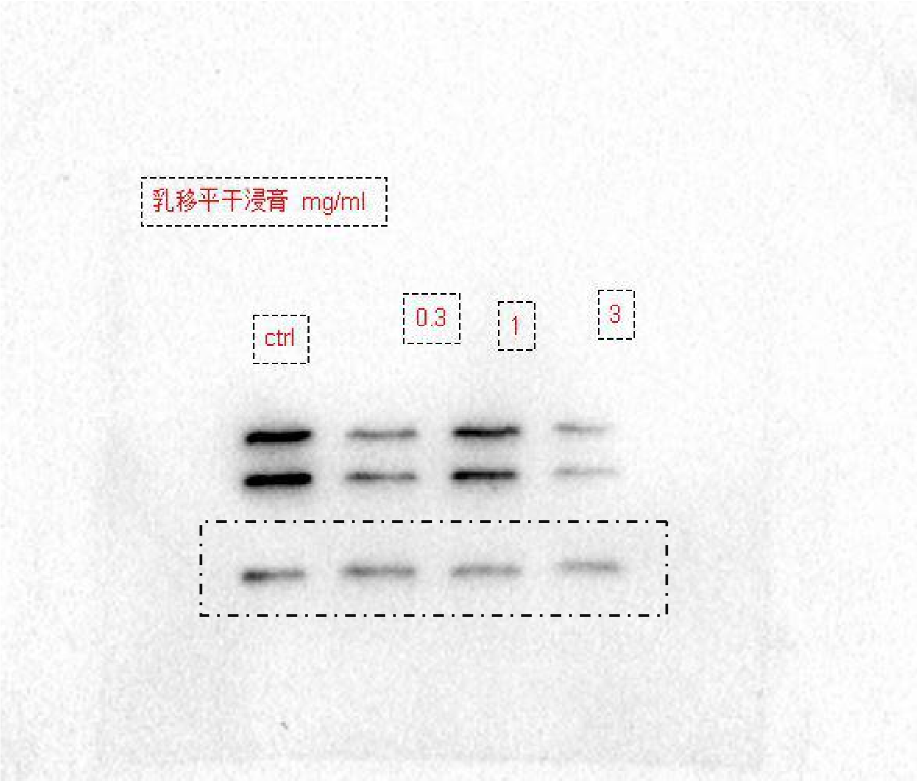

Effects of RYP dry extract on BAX

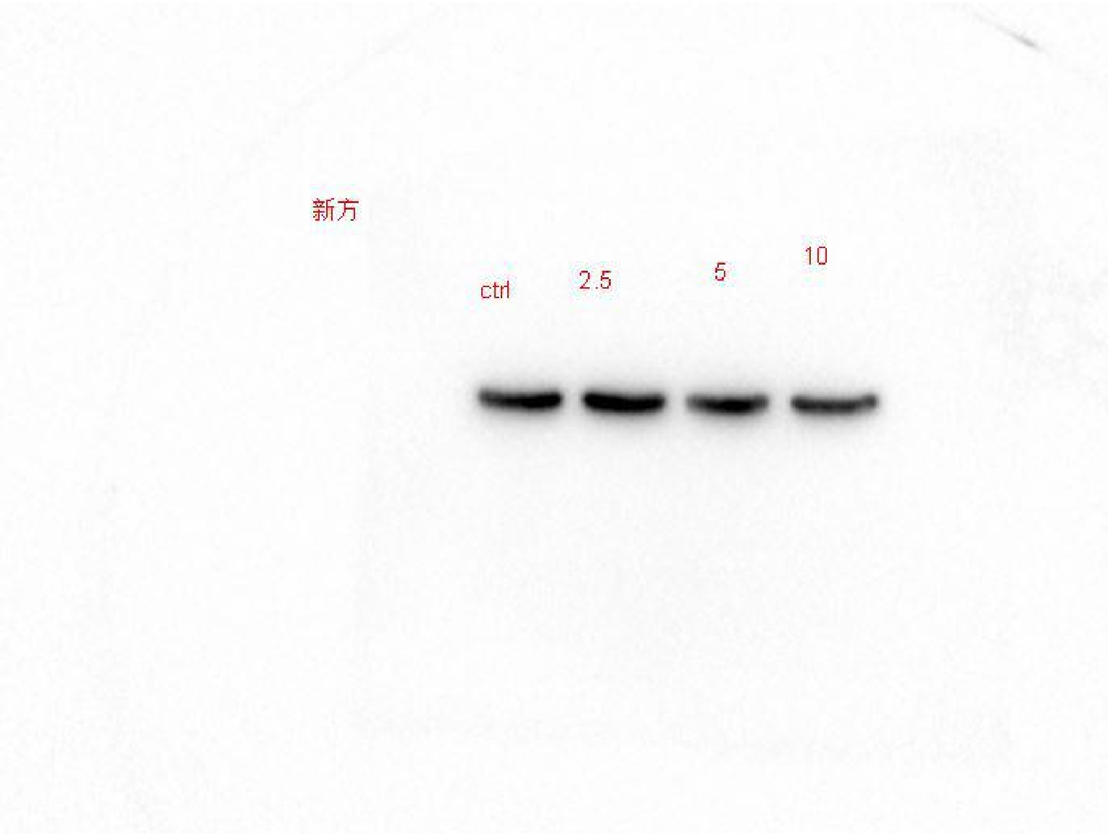

GAPDH control of RYP dry extract on Bcl-2 and Bax

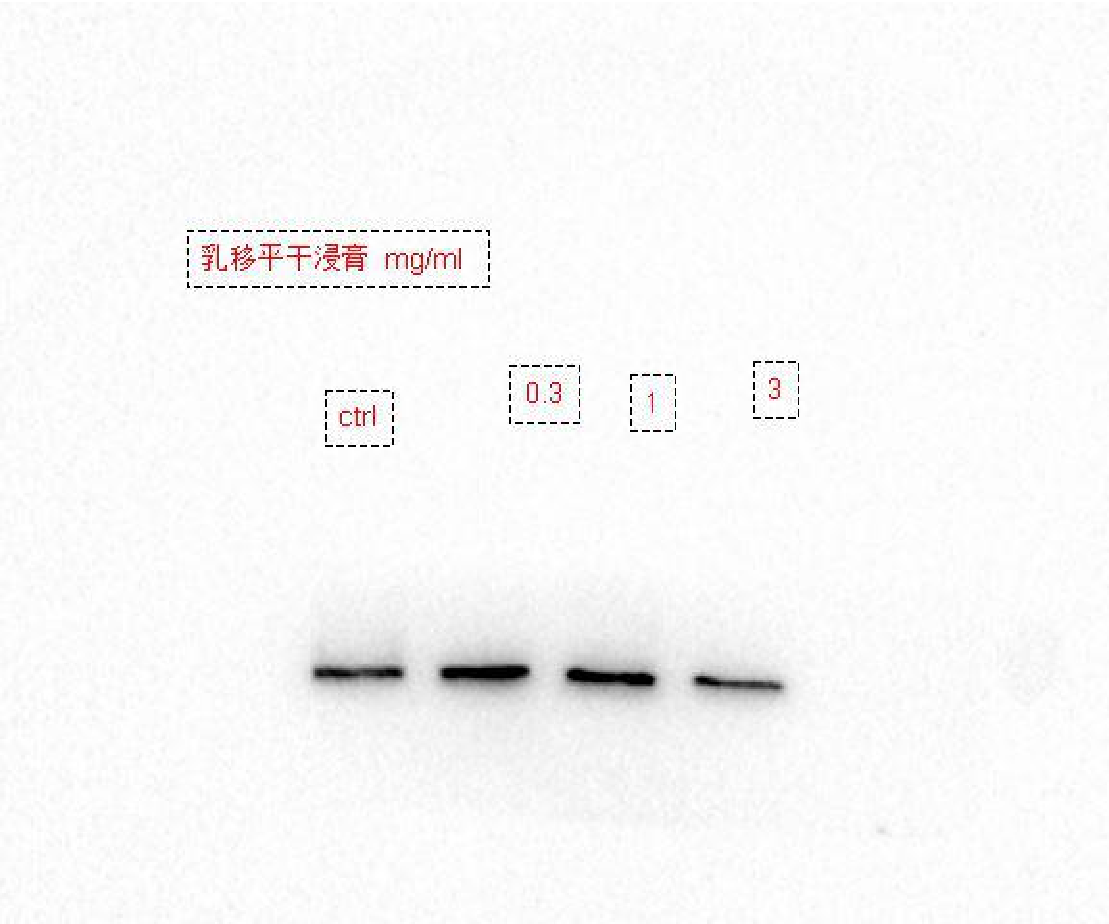

Effects of RYP dry extract on PARP

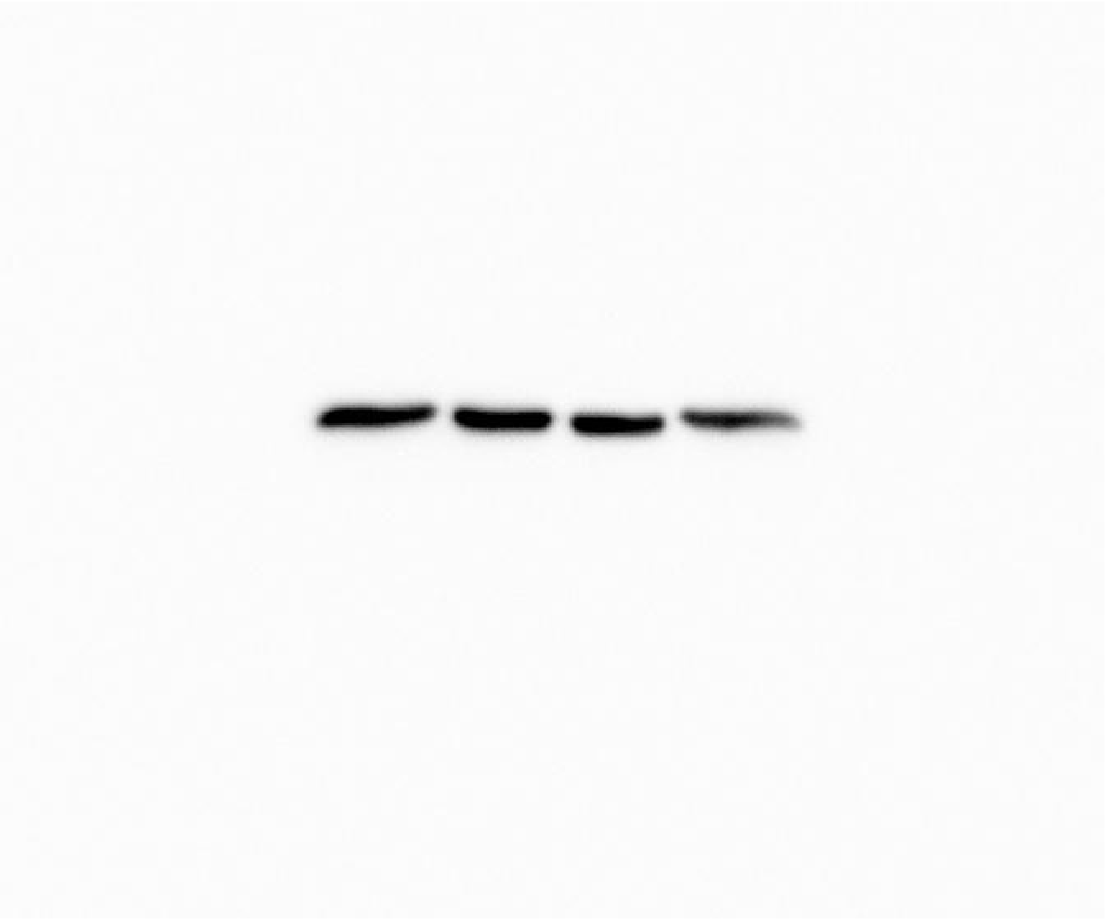

GAPDH control of RYP dry extract of Parp

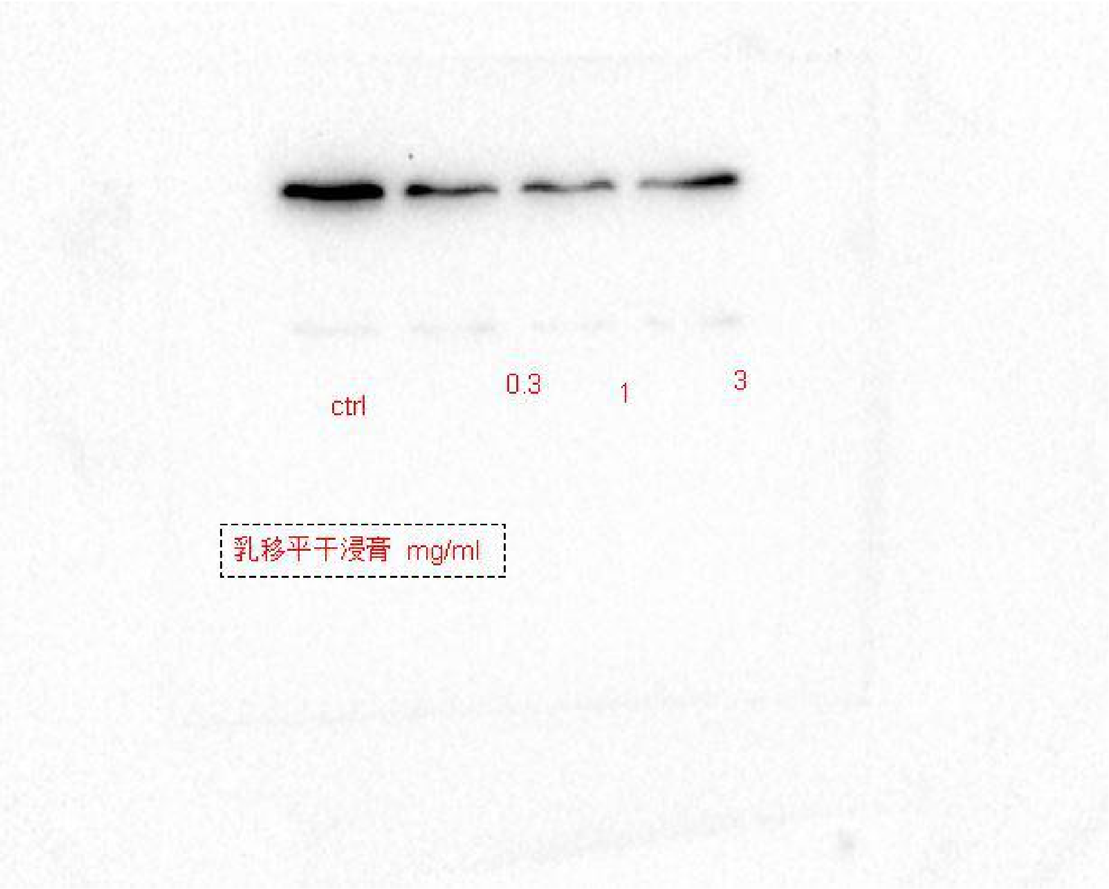

Effects of RYP dry extract on P-akt

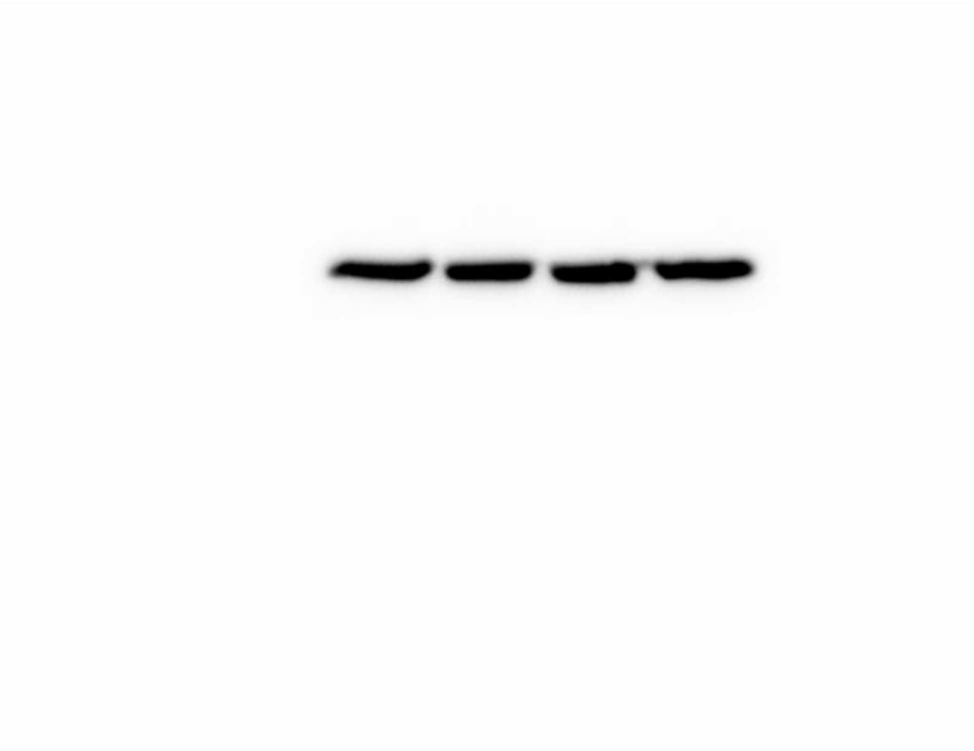

GAPDH control of RYP dry extract of P-akt

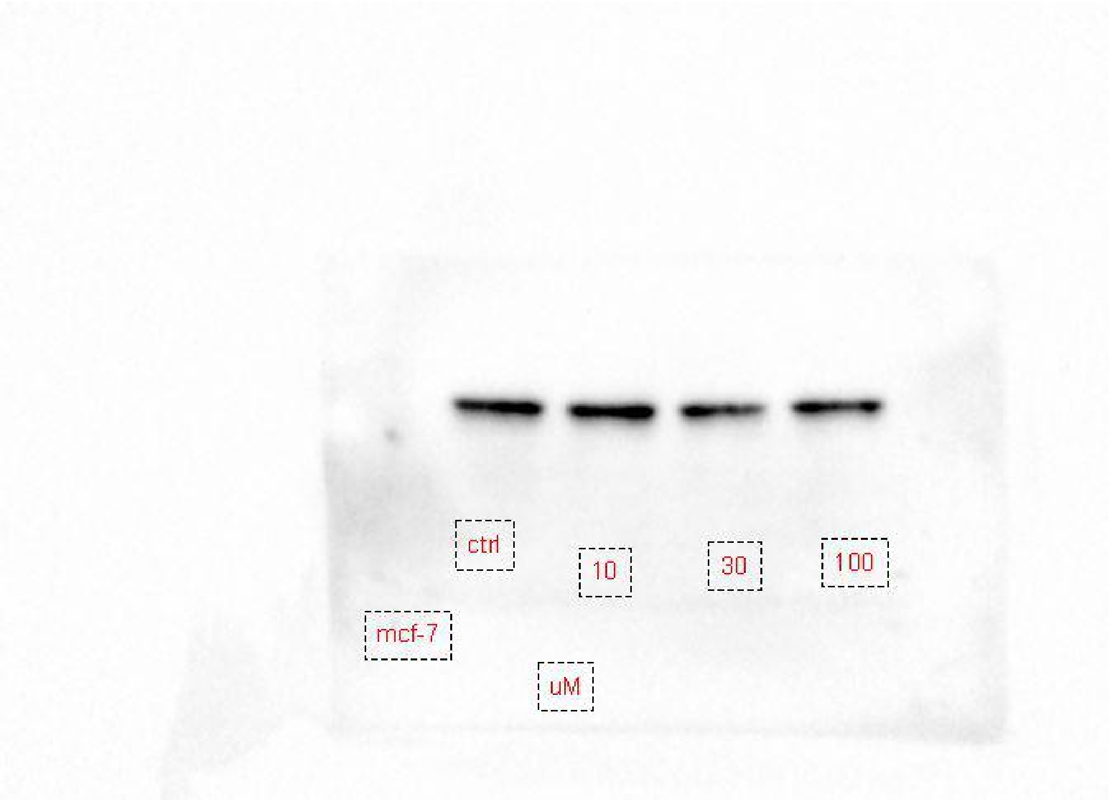

Effects of icariin on BCL-2

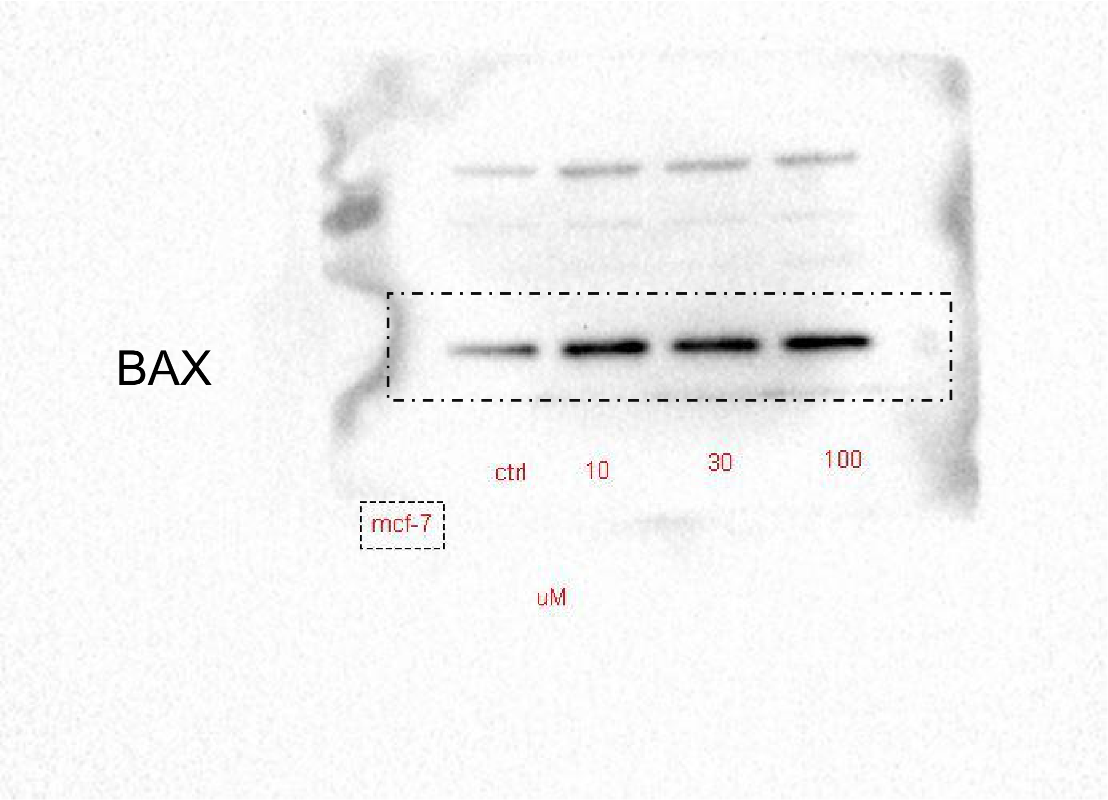

Effects of icariin on BAX

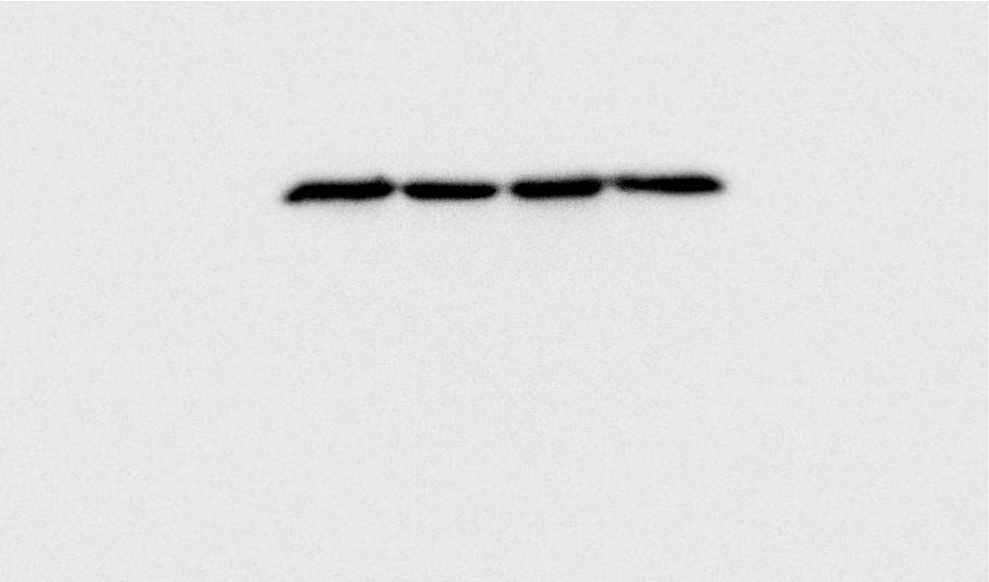

GAPDH control of icariin on Bcl-2 and Bax

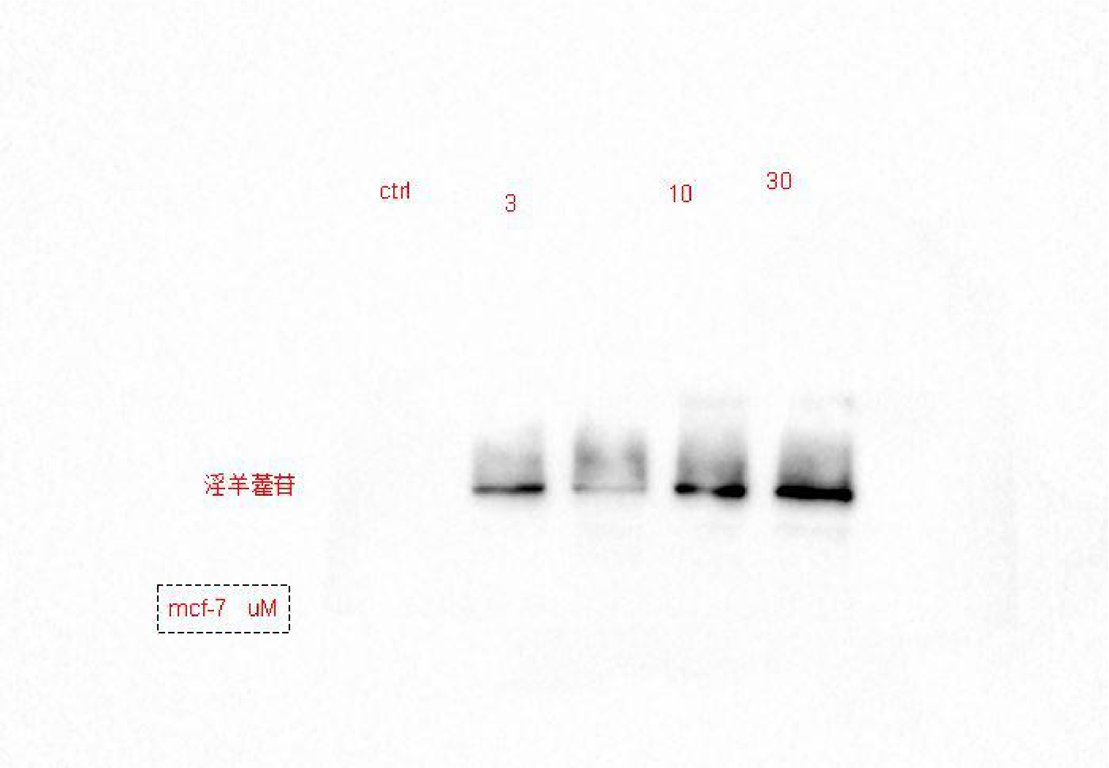

Effects of icariin on PARP

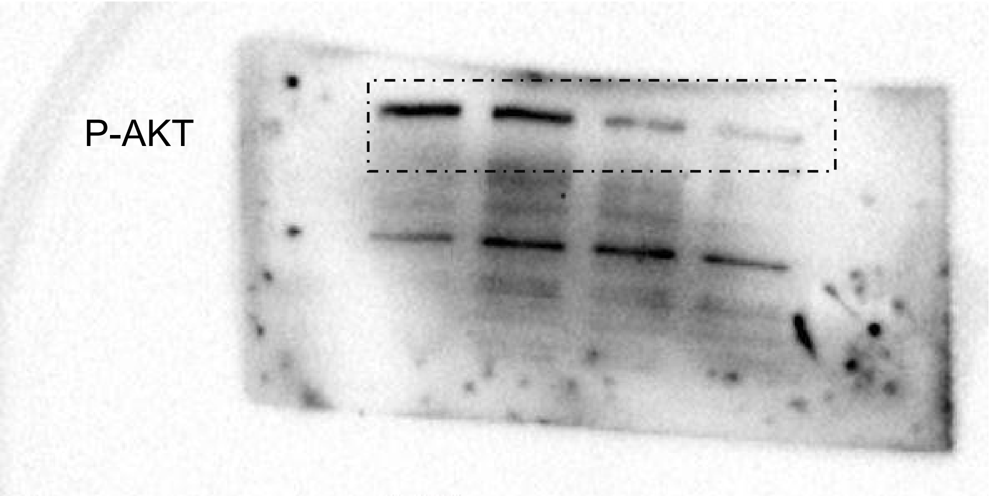

Effects of icariin on P-AKT

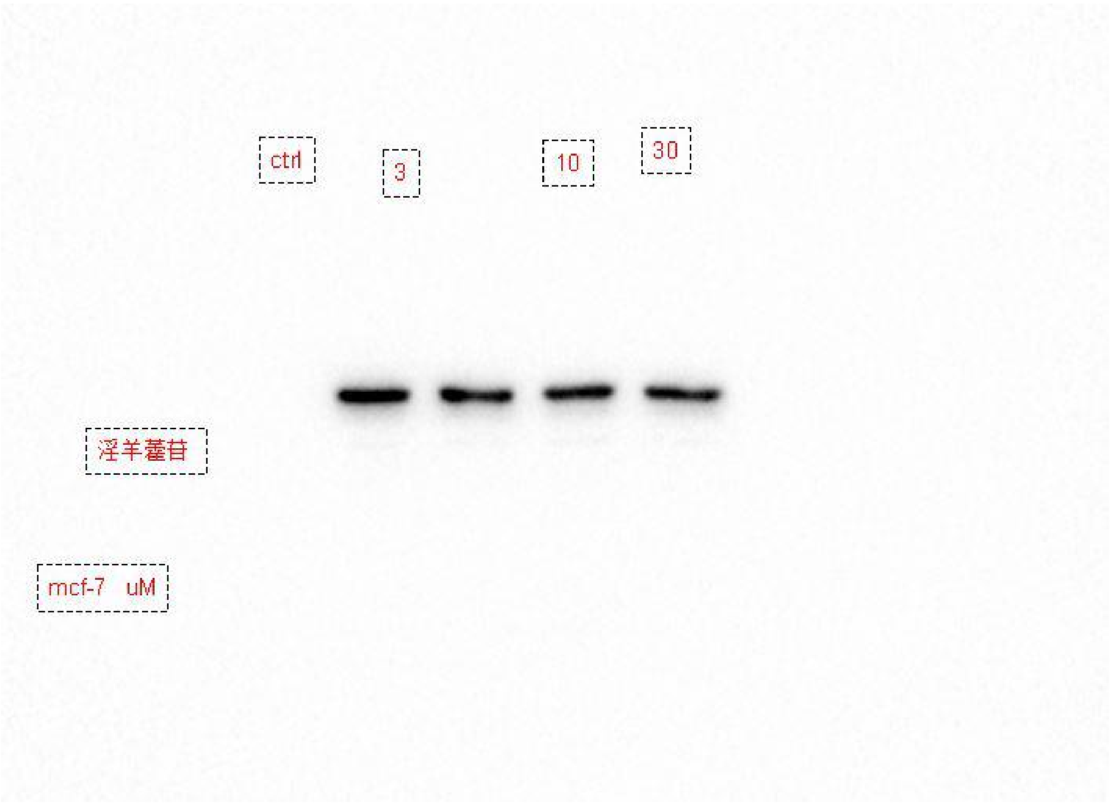

GAPDH control of icariin on Parp and P-akt
